# Quality control method for RNA-seq using single nucleotide polymorphism allele frequency

**DOI:** 10.1111/gtc.12178

**Published:** 2014-09-21

**Authors:** Takaho A Endo

**Affiliations:** RIKEN Center for Integrative Medical Science (IMS-RIKEN)1-7-22 Suehiro-Cho, Tsurumi-Ku, Yokohama, Kanagawa, 230-0045, Japan

## Abstract

RNA sequencing (RNA-seq) provides information not only about the level of expression of individual genes but also about genomic sequences of host cells. When we use transcriptome data with whole-genome single nucleotide polymorphism (SNP) variant information, the allele frequency can show the genetic composition of the cell population and/or chromosomal aberrations. Here, I show how SNPs in mRNAs can be used to evaluate RNA-seq experiments by focusing on RNA-seq data based on a recently retracted paper on stimulus-triggered acquisition of pluripotency (STAP) cells. The analysis indicated that different types of cells and chromosomal abnormalities might have been erroneously included in the dataset. This re-evaluation showed that observing allele frequencies could help in assessing the quality of samples during a study and with retrospective evaluation of experimental quality.

## Introduction

In molecular biological experiments, care must always be taken to prevent contamination from external sources, environmental substances, and undesired cells such as cocultured feeder cells. This has become increasingly important, as transcriptome analysis at the level of the single cell is now more common. Detecting contamination is often very difficult before sequencing experiments, and it is only when results are markedly different from predicted findings that researchers may suspect contamination. Various quality control methods have been proposed, but they focus mostly on other technical aspects of experiments (Wang *et al*. 2012). Here, I describe an approach to detect contaminating cells in studies using next-generation sequencing (NGS), particularly in transcriptome analyses based on RNA-seq techniques.

In RNA-seq experiments, NGS-derived mRNA sequences are compared with the target genome, and aligned reads are collected for all genes for which exon positions are provided in the database. One of the advantages of RNA-seq over microarray techniques is that it also provides some information about genomic sequences.

It is expected that mRNAs originate from both autosomal chromosomes and that the two (or more) alleles would thus be observed in the set of sequence fragments in NGS data. Skew in the frequency of alleles, as assessed by observed SNPs, can indicate several possible biological phenomena. The most preferable source of the skewed distribution, in terms of biological relevance, is allele-specific expression, such as genomic imprinting and mutation. However, another possible source of skewed allele frequencies is sample contamination if the contaminating cells have a different genomic background from genuine target cells.

Although allele frequencies are dependent on the PCR efficiency of each allele, and, in this analysis, variance of the distribution was especially dependent on PCR conditions, the average was approximately 50% for heterozygous SNPs, independent of the cell type.

Allele frequency in RNA-seq has been used to detect imprinted genes (DeVeale *et al*. 2012; Lagarrigue *et al*. 2013). The approach described here extends the application of variation databases for the detection of contaminating cells in RNA-seq studies using heterozygous SNPs. Furthermore, this approach also provides a method for detecting chromosomal abnormalities. Skewed allele frequencies in a specific chromosome can be caused by aneuploidy. As aneuploidy can cause various types of abnormalities, we can exclude data from abnormal cells in studies when aneuploidy is not expected in the target tissue.

This method is applicable for retrospectively evaluating the quality of experiments and is useful for interpreting results that are not apparently reproducible.

## Results and discussion

### Mathematical model and simulation

Diploid cells have pairs of homologous chromosomes, and genes are expressed from the paternal and maternal chromosomes at roughly the same frequency, except in the case of imprinted genes and for genes on sex chromosomes (DeVeale *et al*. 2012; Lagarrigue *et al*. 2013). The frequency of expression of a sequence from a parent is expected by chance to follow a binomial distribution. Because bias caused by PCR amplification can affect this distribution, the influence of PCR bias was examined. When we consider nonimprinting genes having heterozygous SNPs, the allele frequency is expected to be approximately 50% for samples that consist of only one cell type, and the unbalanced representation of certain SNPs caused by contamination should appear as a shift in the distribution peaks.

A simulation was carried out to illustrate how contaminating cells affect the simple binomial distribution of allele frequencies. When the number of the reference allele (A) is *n*_A_ and that of the alternative allele (a) is *n*_a_, the chance of detection of the reference allele is *n*_A_/(*n*_A_ + *n*_a_). In RNA-seq experiments, bias originating from PCR should be considered. To simplify the model, the PCR bias was incorporated by assuming that the sequences containing A and a were amplified 2^α^ and 2^β^ times, respectively. If we obtain N fragments of the locus with RNA-seq, the probability of *k* reference allele sequences is calculated as follows:

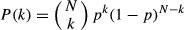


where

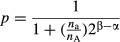


**Figure 1:**
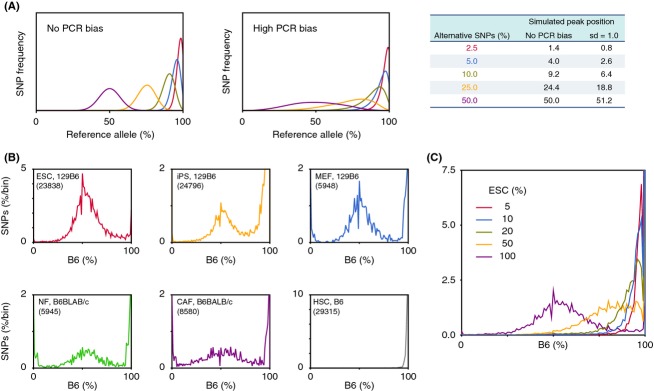
Allele frequency analysis of RNA-seq data. (A) Simulation of SNP allele frequencies using a modified binomial distribution. Peak position was determined by the composition of two alleles, and variance of the distribution was dependent on sd, standard deviation of simulated PCR bias. (B) SNP distributions in several cell types. ESCs (red, SRR1047502, 129B6F1 background), iPSs derived from fibroblasts (yellow, SRR1047504, 129B6F1), MEFs (blue, SRR104220, 129B6F1), normal fibroblasts (NFs; green, SRR1191170, B6 x BALB/c), cancer-associated fibroblasts (CAFs; purple, SRR1191171, B6 x BALB/c), and HSCs (gray, SRR892995, B6). The number of applied SNPs for each cell type is shown in parentheses in each box. (C) Allele frequency of HSC samples contaminated with different percentages of ESCs as shown.

The simulation in Fig.1A was carried out using conditions where *N* = 50 and β − α followed a Gaussian distribution, having standard deviation of 0 (no PCR bias) or 1 (high PCR bias). The simulation indicated that the variance of the distribution was highly dependent on PCR bias and that the mode of the distribution corresponded to the composition of SNP alleles. Allele frequencies of several sets of RNA-seq data from various cell types obtained from public databases were examined, and the results agreed with the simulation (Fig.1B). Peaks at 0 and 100% might result from the homozygous SNPs in observed cells. An artificial contaminating situation was also generated with random sampling of RNA-seq datasets from two cell categories, pure C57BL/6 (B6) hematopoietic stem cells (HSCs), and a mixture of 129 and B6 embryonic stem cells (129B6F1 ESCs) at various ratios. The curve shape and peak positions varied along the ratio as shown in the mathematical simulation (Fig.1C, gray line).

### Re-analysis of STAP paper: Genotype analysis of fibroblast growth factor-induced stem cells (FI-SCs)

**Figure 2:**
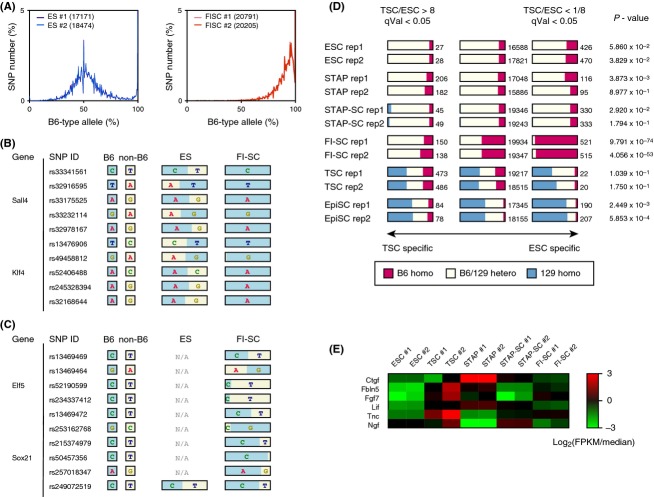
SNPs detected in FI-SC mRNAs indicating contamination. (A) Allele distributions obtained from ESC and FI-SC RNA-seq experiments used in the STAP paper. Both ESCs (blue) and FI-SCs (red) are annotated as having a 129B6F1 genetic background. The number of applied SNPs for each experiment is shown in parentheses in the boxes. (B) SNPs detected in Sall4 and Klf4, which are highly expressed in ESCs. B6-type alleles are shown in blue and 129-type alleles (i.e., non-B6) are in yellow. (C) SNPs detected in the TSC-specific genes Elf5 and Sox21. (D) The number of homozygous/heterozygous SNPs observed in the stem cells used in the original paper. Only the composition observed in FI-SCs would be predicted to affect gene expression. *P*-values were calculated using Fisher's exact test of genotype distribution between TSC-specific genes and ESC-specific genes. Rep1 and rep2 denote two replicated experiments. (E) Heatmap of representative cytokine and extracellular matrix genes that are highly expressed in MEFs. Normalized log ratios of fragments per kilobase of exon per million reads (FPKM) against the medians of all samples were shown.

This study examined how SNP allele frequencies in RNA-seq data can be used to show properties of the dataset. Obokata *et al*. recently reported the phenomenon of STAP, the induced cellular reprogramming of committed somatic cells into pluripotent stem cells that can produce embryonic and placental tissues when injected into blastocysts (Obokata *et al*. 2014a,b). The allele frequency approach described above was used to examine the NGS dataset provided by the researchers. Allele frequencies between reference allele (equivalent to B6 genotype for dbSNP) and alternative allele (corresponding to 129 genotype in this study) were examined in RNA-seq data from seven replicate experiments obtained using the TruSeq reagent (Figs2A and S1 in Supporting Information). The allele distributions of six of the seven experiments showed the equal representation of parental chromosomes expected in Fig.1A. For the experiments that involved ESCs, STAP cells, and STAP stem cells (STAP-SCs), there were no 0% peaks (Fig. S1 in Supporting Information), possibly because the cells were obtained from mice backcrossed in the laboratory that may have a different genotype than those in the public database (J. Sharif and K. Isono, personal communication).

Surprisingly, FI-SCs that were annotated as coming from the F1 129Sv (129) and B6 cell populations did not show the allele distribution pattern of unbiased nonimprinting genes (Fig. S1 in Supporting Information). The distribution was more similar to that of cells with unequal chromosomes. These FI-SCs were reported to be induced from STAP cells with Fgf4 and to have characteristics similar to trophoblast stem cells (TSCs), such as their gene expression profiles and potential to contribute to the placenta (Obokata *et al*. 2014a).

The obvious difference in the FI-SC curve from the 129B6F1 genotype, combined with the fact that the majority of SNPs were similar to B6, suggested that the FI-SCs originated from neonatal mice of a nearly pure B6 background. Further analysis of gene expression patterns suggested that the heterogeneity of SNPs between B6-type allele and non-B6 could be caused by the expression characteristics of genes. As shown in Fig.2B, SNPs expected to be heterogeneous between 129 (i.e., non-B6) and B6 were examined in several ESC marker genes. ESCs carried alleles from both the 129 and B6 backgrounds at these loci, but the FI-SCs, although described as having the same genetic background as the ESCs (Obokata *et al*. 2014a), carried only SNPs from B6. This dominance of the B6 genotype was not observed in TSC marker genes (Fig.2C). The FI-SC specificity was not limited to the genes shown in Fig.2B and C. When all heterogeneous SNPs were classified into three groups, SNPs in ESC-specific genes, SNPs in TSC-specific genes, and SNPs in other genes, only FI-SCs had widely heterozygous SNPs for these groups (Fig.2D). If all included cells in a sample share the same cellular features, one would not expect to see this phenomenon of particular gene sets having different genotypes.

Because the FI-SCs showed a specific genotype at some TSC markers, they may have been contaminated with TSCs. Feeder cells, however, could be another source of contamination, as the FI-SCs were cultured with mouse embryonic fibroblast (MEF) feeder cells whose genotype was not described in the original paper. For this study, the expression of marker genes for MEFs was examined and compared with the expression of ESC and TSC markers, and the results indicated the absence of expression of these MEF genes in FI-SCs (Fig.2E). The probability of contamination by MEFs is therefore negligible, and the most likely explanation for the skewed distribution of allele frequencies detected in the duplicated RNA-seq experiments is that the FI-SC population originated from two cell types: ESC-like cells having a B6 genetic background and TSC-like cells having a genotype similar to that of CD1, which is a mouse strain other than B6 and 129.

### Re-analysis of a retracted paper: Detection of chromosomal aberrations

**Figure 3:**
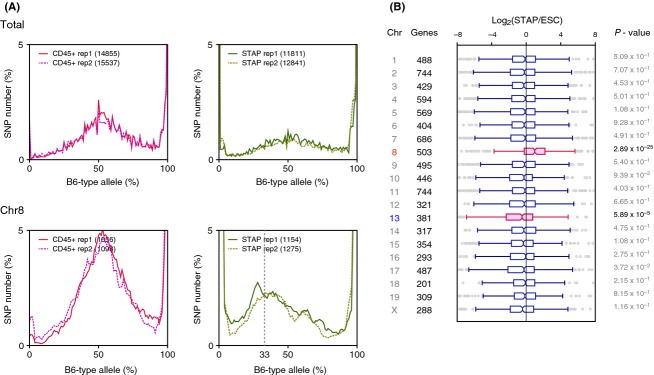
Trisomy detected by SNP analysis of RNA-seq data. (A) Allele frequency distributions of whole chromosomes and chromosome 8. Only chromosome 8 of STAP cells had a peak that was not centered approximately 50%, indicating that chromosome 8 originating from the 129 strain was duplicated to produce trisomy of the chromosome. Unlike the RNA-seq data analyzed in Figs1 and 2, the RNA-seq data analyzed in this figure were generated using the SMARTer reagent kit. (B) Expression analysis by chromosome. Only genes on chromosome 8 were significantly more expressed, and genes on chromosome 13 were significantly less expressed. *P*-values were calculated using two-sided Student *t*-tests.

SNP distribution analysis can also be applied to detect aneuploidy. In examining allele frequencies for each chromosome, abnormal chromosomes are assumed to have skewed distributions when paired chromosomes do not have the equal number of duplicates. As shown in Fig.3A, chromosomal analysis showed abnormality of chromosome 8 in the STAP cells used in the original study. We can expect the peak allele frequency to occur at approximately 50% if a cell has two chromosomes from parents of different strains. The peak allele frequency of the STAP cells, however, was at approximately 33%, meaning that one of the two chromosomes appears to be duplicated leading to three copies of chromosome 8. The STAP cells used in the experiments were derived from 129 and B6 cells, and the duplicated chromosome is presumed to be from the 129 parent because it contained only non-B6 SNP alleles. It is notable that trisomy 8 is the most common chromosomal aberration in mouse ESCs, with 31 of 97 examined cell lines reported to carry this abnormality (Mayshar *et al*. 2010). ESCs having trisomy 8 have a growth advantage, but chimeras will not transmit the mutation to the germ-line (Ben-David *et al*. 2013), and trisomy 8 in mice results in prenatal death by day 12 or 13 (Kim *et al*. 2013).

Because aneuploidy detection using transcriptome analysis has been reported (Gropp 1982; Liu *et al*. 1997), the expression of genes on each chromosome was analyzed in this study. Genes on chromosome 8 and chromosome 13 had significantly different expression patterns between STAP cells and ESCs. Chromosome 8 gene expression was 1.3 times higher in STAP cells than in ESCs (*P*-value = 2.89 × 10^−25^; Fig.3B). This result is concordant with trisomy of chromosome 8 as detected by SNP analysis. The SNP allele frequency method used here can, therefore, be used to detect aneuploidy if we know the SNP genotype of the cells and if the control cells have a normal karyotype.

### Availability of SNP allele frequencies

The SNP allele frequency method detected contaminating cells in samples used to generate RNA-seq data, but the sensitivity of this method is dependent on the depth of sequence reads. Based on the limited number of RNA-seq datasets here, the graphs become too noisy to detect differences in the distributions if the number of available SNPs is fewer than approximately one thousand. For example, MEF cells having only 5948 SNPs generates a more noticeable amount of spikes than data for ESCs with 23 838 SNPs. SNPs assigned to each chromosome also suggested that the distributions could be very noisy when the number of SNPs become <1000 (Fig. S2 in Supporting Information). Cover ratios are also important for the sensitivity because the variance of binomial distribution depends on the number of trials. Therefore, when the average cover ratio is required to be 20× for all genes, the required read count will be approximately 1.3 × 10^9^ nucleotides, roughly corresponding to a read count of approximately 1.1 × 10^9^ bases, which was used to derive the distribution for MEFs (Fig.1B).

Figure1B also indicates some interesting aspects of genome stability of induced pluripotent stem (iPS) cells. The iPS cells generated from 129B6F1 had more homozygous SNPs of B6-type alleles. Although, as noted earlier, this may be the result of cellular contamination or this could be the result of differences in the properties of the cells used in the two experiments, there is also the intriguing possibility that the experimental process induced a transition of genotypes. As iPS cell engineering has been reported to induce genomic and/or epigenomic instability (Hussein *et al*. 2011; Chang *et al*. 2014), it will be important to examine allele frequencies of iPS cells in future studies.

SNP frequency differences detected in RNA-seq or other NGS experiments are probably not more accurate than those detected by direct observation of karyotypes; however, the method described in this study can be used to detect chromosomal abnormalities even if the test cells are no longer available, and from both genotype (SNPs) and phenotype (gene expression), which is expected to provide more reliable evidence than genotype or phenotype alone.

One advantage of the SNP allele frequency method of this study is its potential ability to detect and determine the parental origin of chromosomes duplications. The other advantage over virtual karyotyping (Ben-David *et al*. 2013) is that it does not require control cells without aneuploidy because simulated models expect the allele frequencies of diploid cells would have peaks approximately 50% (Fig.1A).

### Contamination in FI-SC samples

Examination of allele frequencies in RNA-seq fragments enables detection of specific characteristics of the sampled cells. When a set of genes specifically expressed in a particular cell type shows a distinct genotype, the origins of the cells can be assumed. This analysis of SNPs in mRNA sequence reads from the Obokata *et al*. study showed that the origins of the STAP and FI-SC samples were likely different than originally reported. The simulation showed that the mode of the distribution depends both on the cellular composition of the sample and possible variance caused by PCR bias. When we ignore the PCR bias of binomial distribution, the ratio of contaminating cells can be roughly estimated by the peak position in the distribution.

**Figure 4:**
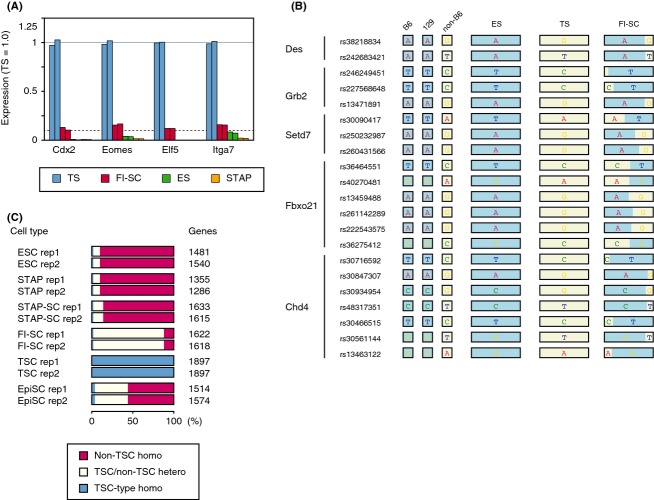
Examination of incorporated mRNAs in FI-SCs. (A) Expression of TSC marker genes in ESCs, TSCs, and FI-SCs. The solid line indicates the average TSC gene expression and dashed line indicates 10% of the average. (B) Heterozygous SNPs detected in Des, Grb2, Setd7, Fbxo21, and Chd4 where B6 and 129 share the same SNPs but TSCs did not. (C) Gene-wise analysis of distribution of heterozygous/homozygous SNPs having TSC-specific alleles.

The most likely cell type to be contaminating the FI-SCs in the original study, TSCs, had many more heterozygous (B6/non-B6) alleles than non-B6 homozygous alleles. SNPs of FI-SCs were counted in alleles where B6 and 129 have different nucleotides and duplicated experiments resulted in 6859 and 7243 heterozygous SNPs and 24 and 14 non-B6 homozygous alleles, respectively. The percentage of contaminating cells can be assumed with genotype observation because the peak allele frequencies were at approximately 95–96%, whereas the population peak is expected to be at approximately 10%. This result was concordant with the expression of TSC marker genes. All of the examined TSC marker genes examined were expressed in approximately 10% of TSCs (Fig.4A).

FI-SC-specific SNPs were also examined where B6- and 129-derived sequences shared the same nucleotide but FI-SC-derived ones did not. Most of these SNPs matched with the CD1 background of the TSCs used in the experiment and were different from B6 or 129. Gene-by-gene representation (Fig.4B) and whole-SNP analysis (Fig.4C) showed that FI-SCs shared TSC-specific SNPs. As the proportion of contaminating TSCs in the FI-SCs was minor, most of the TSC-specific SNPs appeared heterozygous. These results support the view that the RNA-seq data contained transcripts from two major cell populations, with approximately 90% B6 cells having an ESC-like expression pattern and approximately 10% CD1-like cells having a TSC-like pattern. Therefore, the claim in the Obokata *et al*. paper that FI-SCs contribute to the placenta might have been based in error on contamination by TSCs, which are known to be organ stem cells (Tanaka *et al*. 1998).

### Interpretation of the STAP phenomenon

Detection of aneuploidy using SNPs also suggested contamination in the original experiments. Both genotype and phenotype analyses suggested that the STAP cells used in the Obokata *et al*. experiments had trisomy of chromosome 8, and a transcription assay indicated atypical expression of genes on chromosome 13. Trisomy 8 is the most common chromosomal abnormality in mice, and chromosome 8 has been reported to fuse to the end of chromosome 13 (Kim *et al*. 2013). These observations might explain the results of virtual karyotyping in Fig.3B. The RNA-seq data used in Fig.3 were annotated as being derived from neonatal mouse spleen cells cultured in conditions under which the STAP cells did not grow. This description does not agree with the dominance of cells having trisomy because mice carrying pure trisomy 8 are embryonic lethal. This therefore leads to the conclusion that the cells were cultured cells that possessed expression characteristics that were very similar to those of ESCs.

## Conclusion

The SNP allele frequency method described here is limited by the fact that if the contaminating and examined cells share a common genetic background, the allele frequencies will not be different enough to detect the contamination. However, this method is, in principle, applicable to any RNA-seq data that contain polymorphisms and will be useful for both prospective and retrospective quality control of RNA-seq experiments, especially for studies using cultured cells such as ESCs and iPS cells and their derivatives.

## Experimental procedures

### Dataset

Mouse variation data were obtained from the Sanger Mouse Genomes Project (http://www.sanger.ac.uk/resources/mouse/genomes/), and the version 137 VCF-formatted dataset was retrieved from dbSNP (http://www.ncbi.nlm.nih.gov/SNP/).

The locations of genes and included exons were obtained from iGenomes (http://support.illumina.com/sequencing/sequencing_software/igenome.ilmn). SNPs outside exons were excluded from the original VCF file using this annotation, and thus, 1 016 227 SNPs were used for the entire dataset.

Raw sequencing data from the original RNA-seq experiments examined in this study were downloaded from the short read archive (SRA) at NCBI. The accession number of the project is SRP038104. Genome sequences of mouse (version 38, mm10) obtained from B6 mouse strain were downloaded from NCBI GenBank and encoded into a bowtie database using the bowtie-build (for colored space fastq files) or bowtie2-build (for fastq files) program. The accession numbers (i.e., SRA ID) of the RNA-seq experiments are listed in Table S1 (Supporting Information), and the checksums of archived sequences were confirmed by Dr Teruhiko Wakayama, Yamanashi University, one of the corresponding authors of the original paper.

### RNA-seq analysis

The SRA database sra-format files were converted into fastq format using sratoolkit.2.3.4-2. The Bowtie2 (version 2.1.0, http://bowtie-bio.sourceforge.net/bowtie2/index.shtml) and tophat2 (http://tophat.cbcb.umd.edu) programs were applied for sequence alignment. Because this study did not consider the structure of the mRNAs, all sequences were fragmented into 50-bp reads and aligned using tophat or tophat2 with the parameter “–no-coverage-search -G genes.gtf” allowing two mismatches. The tophat program was used only to analyze SOLiD colored space fastq files. Levels of gene expression were evaluated with fragments per kilobase of exon per million reads (FPKM) values calculated using cufflinks (version 2.1.1). A program written in C++ was developed to detect and enumerate SNP alleles in BAM files. The program is open-source software available in a public repository (https://github.com/takaho/snpexp/).

### SNP identification and heterozygosity tests

Sequence fragments aligned on the mouse genome were analyzed using the SNP detection and enumeration program mentioned above, and only SNPs with cover ratios ≥20 were retained. If ≥95% of the SNP sequence was the same on the two strands, the allele was designated as homozygous.

Genomewide heterozygosity between B6 and 129 was analyzed using a subset of the SNPs described above with different alleles between B6 and 129. SNPs were classified by known expression characteristics (TSC-specific, ESC-specific or other) and genotypes (B6-type homozygous, 129-type homozygous or other). SNP distributions were tested with *P*-values obtained using Fisher's exact test with Monte-Carlo Markov chain approximation.

### Expression analysis by chromosome to identify trisomy

FPKM values originating from ESCs (SRR1171574 and SRR1171575) and STAP cells (SRR1171578 and SRR1171579) were calculated, and genes having >0.01 FPKM in all four original experiments were selected. Genes without pseudogenes were classified for chromosomes, and the log ratio of the average of two experiments using the same cells was determined for each chromosome. The distribution of relative FPKM values was evaluated using one-sided *t*-test against the mean log ratio of whole genes.

### MEF marker genes

Marker genes of feeder cells were identified using unpublished RNA-seq data provided by Dr Jafar Sharif and Dr Kyoichi Isono. Gene expression differences among ESCs, TSCs and MEFs were compared using the cuffdiff program, and genes that were significantly highly expressed in MEFs were selected. Genes encoding cytokines and extracellular matrix-related genes were selected to illustrate the features of feeder cells in Fig.2E.

## Supporting Information

**Figure S1** Allele distributions from the RNA-seq data obtained for the cell lines reported in the Obokata. study. CD45^+^ cells (gray), ESCs (yellow), STAP cells (blue), STAP-SCs (green), TSCs (orange), EpiSCs (light blue), and FI-SCs (red). The ESCs, STAP cells, STAP-SCs, FI-SCs, and epiblast stem cells (EpiSCs) were annotated as being derived from a 129B6F1 strain, and the TSCs as from a CD1 strain.

**Figure S2** Allele frequencies of all chromosomes. SNPs on all autosomes and the X chromosome were counted, and their distributions are indicated. The RNA-seq data from the CD45^+^ and STAP cells are identical to those used in Fig.3. Numbers after each chromosome name are those of applied SNPs of CD45+ rep1, CD45+ rep2, STAP rep1, and STAP rep2, respectively.

**Table S1** RNA-seq raw data used in this study
