# Supplementary figures and images for: Quality control method for RNA-seq using single nucleotide polymorphism allele frequency
Source: Genes Cells. 2014 Sep 21;19(11):821–9. doi: 10.1111/gtc.12178 (PMC4231238; doi:10.1111/gtc.12178)

Figure S1

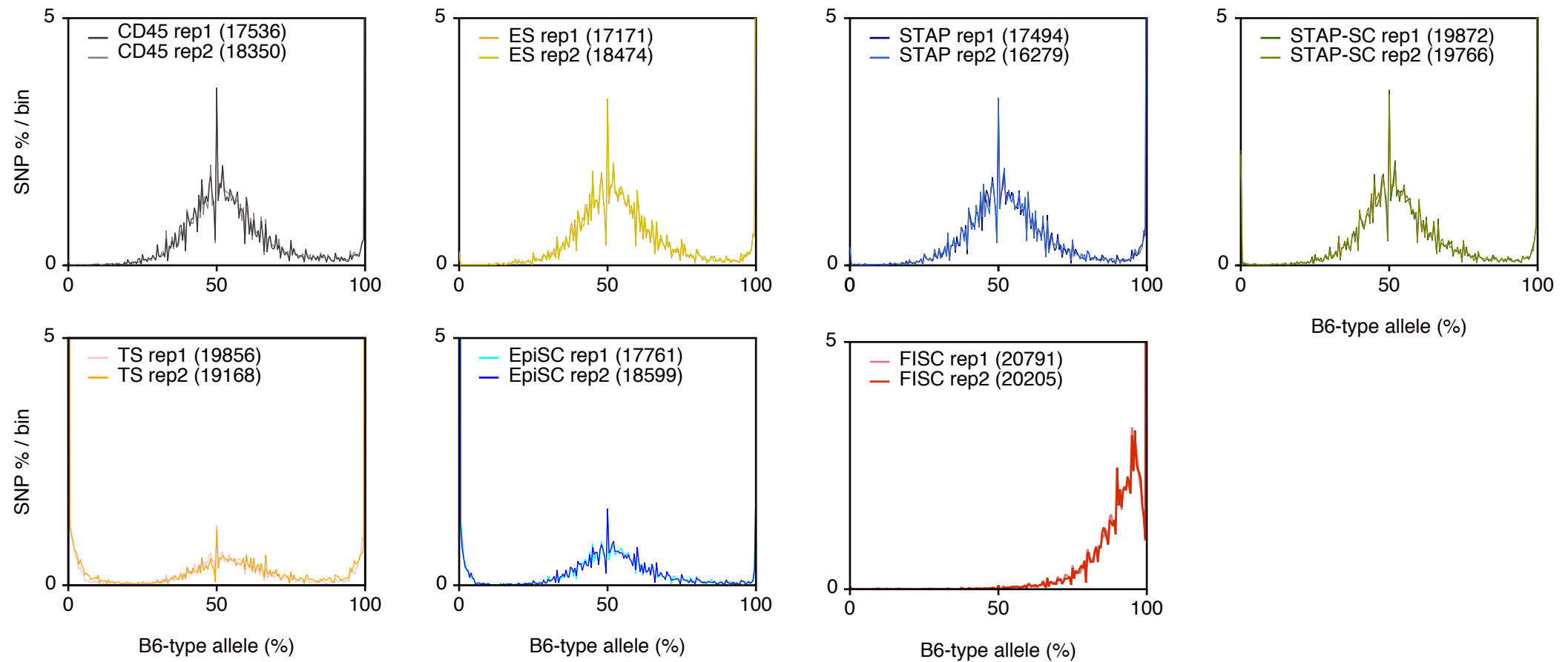

Supplement: Supplementary file 1 — Figure S1 Allele distributions from the RNA-seq data obtained for the cell lines reported in the Obokata. study. CD45+ cells (gray), ESCs (yellow), STAP cells (blue), STAP-SCs (green), TSCs (orange), EpiSCs (light blue), and FI-SCs (red). The ESCs, STAP cells, STAP-SCs, FI-SCs, and epiblast stem cells (EpiSCs) were annotated as being derived from a 129B6F1 strain, and the TSCs as from a CD1 strain. [file gtc0019-0821-sd1.pdf]

Figure S2

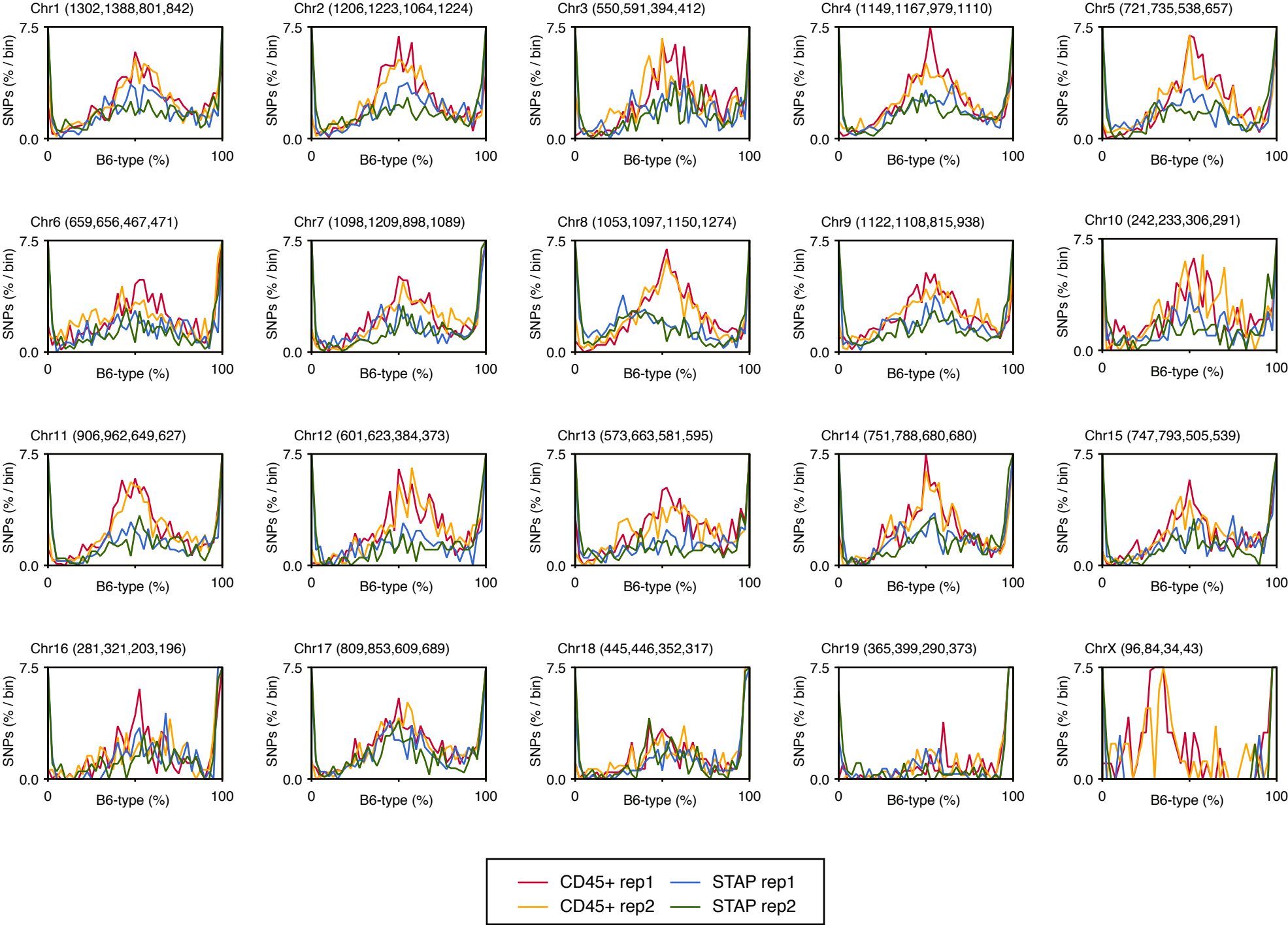

Supplement: Supplementary file 2 — Figure S2 Allele frequencies of all chromosomes. SNPs on all autosomes and the X chromosome were counted, and their distributions are indicated. The RNA-seq data from the CD45+ and STAP cells are identical to those used in Fig.3. Numbers after each chromosome name are those of applied SNPs of CD45+ rep1, CD45+ rep2, STAP rep1, and STAP rep2, respectively. [file gtc0019-0821-sd2.pdf]
